# DNA Nanoflowers’ Amelioration of Lupus Symptoms in Mice via Blockade of TLR7/9’s Signal

**DOI:** 10.3390/ijms232416030

**Published:** 2022-12-16

**Authors:** Jing Wang, Mingzhe Gan

**Affiliations:** 1 Laboratory Animal Center of Soochow University, Suzhou 215123, China; 2 CAS Key Laboratory of Nano-Bio Interface, Suzhou Institute of Nano-Tech and Nano-Bionics, Chinese Academy of Sciences, Suzhou 215123, China

**Keywords:** DNA nanoflower, inhibitory ODN, TLR7, TLR9, antagonists, systemic lupus erythematosus (SLE)

## Abstract

Inhibitory oligodeoxynucleotides (INH-ODN) can exert an immunomodulatory effect to specifically block TLR7 and TLR9 signaling in systemic lupus erythematosus (SLE). To extend the half-life of INH-ODN in vivo, the phosphorothioate backbone, instead of the native phosphodiester, is preferred due to its strong resistance against nuclease degradation. However, its incomplete degradation in vivo may lead to potential risk. To solve these problems and enhance the blockage of TLR7 and TLR9, we prepared highly compressed DNA nanoflowers with prolonged native DNA backbones and repeated INH-ODN motifs. Three therapeutic types of nanoflower, incorporating INH-ODN sequences, including IRS 661, IRS 869, and IRS 954, were prepared by rolling circle amplification and were subcutaneously injected into MRL/lpr mice. The TLR7 blocker of the IRS 661 nanoflower and the TLR9 antagonist of the IRS 869 nanoflower could decrease autoantibodies, reduce cytokine secretion, and alleviate lupus nephritis in mice. However, the IRS 954 nanoflower, the TLR7 and TLR9 dual antagonist, did not have additive or opposing effects on lupus nephritis but only showed a decrease in serum IFNα, suggesting that the TLR7 and TLR9 antagonist may have a competition mechanism or signal-dependent switching relationship. INH-ODN nanoflowers were proposed as a novel and potential therapeutic nucleic acids for SLE.

## 1. Introduction

Systemic lupus erythematosus (SLE) is an autoimmune disease. Susceptible individuals can be triggered by environmental or infectious factors to induce the activation of T and B lymphocytes, production of autoantibodies, and deposition of immune complexes in tissues and organs. Lupus nephritis is a common and severe complication of SLE, which may quickly lead to end-stage renal disease and even death [1]. Therefore, studying the pathological mechanism and treatment strategy of lupus erythematosus has become urgent.

Toll-like receptors (TLRs) are a class of very important nucleic acid sensors that activate cellular functions by recognizing specific nucleotide sequences contained in the CpG oligonucleotides of bacteria and other prokaryotes to activate pDC. Chloroquine is currently commonly used in the clinical treatment of SLE to inhibit TLR signaling. However, its application is limited due to its obvious side effects [2]. Therefore, specific inhibition of TLRs is considered a strategy for treating SLE. TLRs are mainly expressed in B cells, plasmacytoid dendritic cells (pDCs), and macrophages and regulate innate and acquired immunity. In particular, TLR7 and TLR9 can bind to immune complexes with ssRNA antibodies or dsDNA autoantibodies in patients, respectively [3]. The endocytosis with a B-cell receptor (BCR) in autoreactive B cells binds to DNA-associated antigens (such as cytosine–phosphate–guanosine (CpG)) and RNA-associated antigens helped by type I interferon. These autoantigens at the surface of B cells can be internalized into endosomes, recognized by the BCR, and then trigger TLR7 and/or TLR9 [4]. This process increases the level of interferon α (IFN-α), promoting the differentiation of plasmacytoid DCs (pDC) and the expression of the MyD88 protein [3,5]. Then, pDCs produce a large amount of type I interference factors, especially IFN-α, causing immune activation [6]. The SLE-associated cytokine environment determines the pathogenic B-cell response to TLR7/9 [7]. Rommler found that agonists of TLR7 or TLR9 activated pDCs in lupus patients, even in healthy people, to induce a large amount of IFNα and immune overactivation. Thus, treatment of TLR7/9 inhibitors could be a therapeutic strategy for lupus [8].

Oligodeoxynucleotides (ODNs) can exert immunomodulatory effects. Inhibitory ODNs (INH-ODN) that adjust CpG motifs or backbones antagonize TLR signaling, thereby down-regulating inflammatory responses and reducing inflammatory responses. In addition, some specific immunosuppressive ODNs confirm the antagonism of TLR7 or TLR9. However, to resist nuclease degradation, the native phosphodiester (pO) in INH-ODN is always replaced by the phosphorothioate (pS) backbone, which leads to unclear biocompatibility and potential risk. Furthermore, previous studies have found that the effect of INH-ODN is not only related to its specific sequence but also may depend on the length of the backbone and the spatial structure formed by three-dimensional folding [9,10].

TLR7/9 are mostly expressed in pDCs and B cells, and both share the same downstream signaling pathway, i.e., the myeloid differentiation factor 88 (MyD88) pathway. Although the roles of TLR7 and TLR9 have been investigated recently, the difference in their effects when inhibiting TLR7 and TLR9 is still unclear. Generally, TLR7 is considered to bind to ssRNA, guanosine analogs, and synthetic imidazoquinoline-like molecules. At the same time, TLR9 recognizes CpG DNA motifs and DNA strands from bacteria and viruses. When TLR7 or TLR9 bind to their ligands alone, the expression of MyD88 up-regulates, and the activation of MAPKs and inflammatory transcription factors such as IRF-7, NF-κB, and AP-1 are induced. The MAPKs pathway mediates the maturation of DCs and regulates immune responses [11]. However, the immune-regulation effects of the combination of TLR7 and TLR9 blockades remain to be studied.

In this paper, the reported TLR7 blocker, named IRS 661 [10]; the TLR9 antagonist IRS 869 [12]; and the dual inhibitor of both TLR7 and TLR9, named IRS 954 [13], were selected. We prepared a novel DNA nanopolymer via rolling circle amplification. The DNA nanopolymer containing repeat target sequences was compressed into many spatial folds with a long backbone. This nanopolymer, called a nanoflower, was made of natural DNA, which avoided biosafety risks due to DNA’s native safety. The prepared DNA nanoflower was highly compressed into a nano-scale diameter with multiple favorable factors for ligand binding. We predicted that the prepared DNA nanoflowers could enhance the immunosuppressive effect in lupus-prone mice. Meanwhile, the treatment results of the dual inhibitor of TLR7 and TLR9 were compared with the sole antagonist against TLR7 or TLR9 alone.

## 2. Results

### 2.1. Characterization of the Prepared DNA Nanoflowers

**Figure 1:**
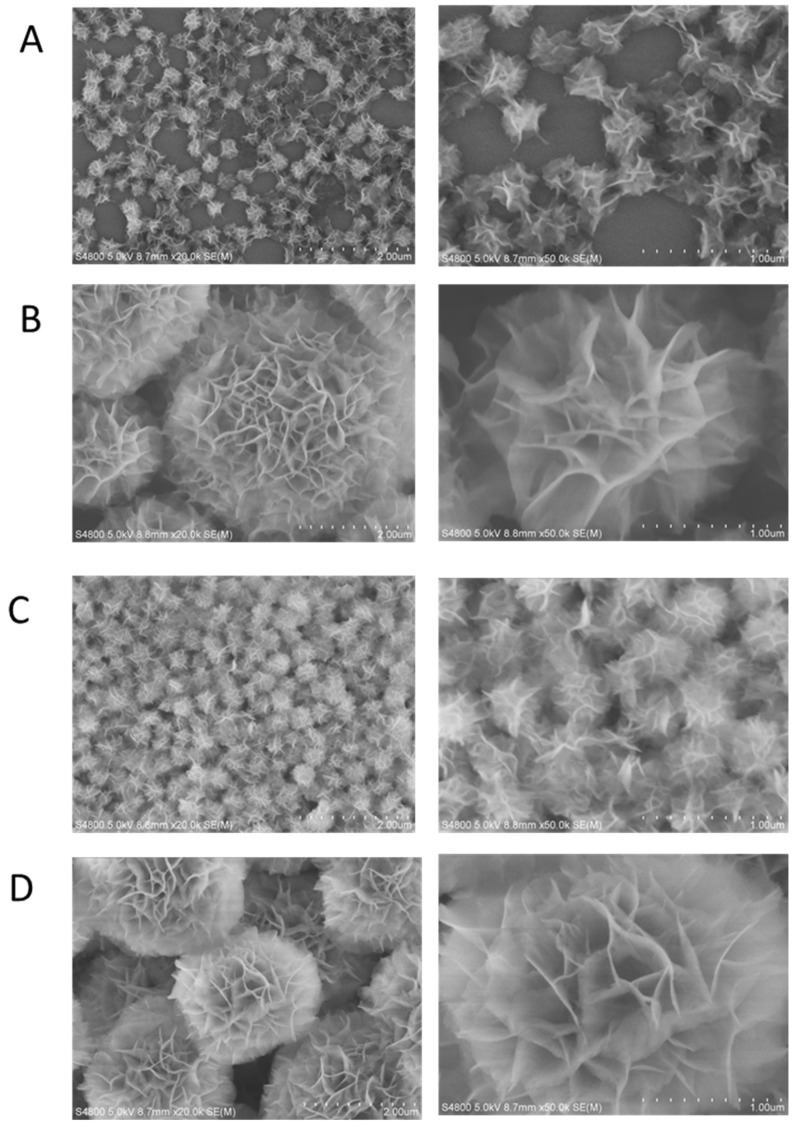
Microphotograph of nanoflower (magnification: left × 20,000, right × 50,000). (**A**). Prepared IRS 661 DNA nanoflower; (**B**). prepared IRS 869 DNA nanoflower; (**C**). prepared IRS 954 DNA nanoflower; (**D**). prepared CTR DNA nanoflower.

The sequences of IRS 661, IRS 869, IRS 954, and CTR were synthesized by multiple rolling circle amplification to form nanoflowers. The final product was intertwined, long-chain ssDNA. It was entangled into a three-dimensional network structure. Its free ends were entangled and folded multiple times. Finally, a tangled “flower-like” or “hair-ball like” shape was formed, called a DNA nanoflower. The characteristics of prepared IRS 661 nanoflower, IRS 869 nanoflower, IRS 954 nanoflower, and CTR nanoflower are shown in Figure 1 via scanning electron microscope. The results showed that DNA nanoflowers were successfully prepared with a nanometer-scale diameter. The free ssDNA ends were paired with each other. The exposed backbone and the target sequence showed different two-dimensional and three-dimensional structures.

### 2.2. Body Weight and Organ Indexes

The main organs of every mouse were immediately collected for weighing and analysis after 14 weeks of continuous treatment. The results are shown in Table 1. In comparison, the IRS-661-nanoflower-treated group, the IRS-869-nanoflower-treated group, and the CTR-treated group showed heavier body weight (compared with the model group, *p* > 0.05). The liver weight of all the nanoflower treatments was higher than that in the model group (*p* > 0.05).

The results of the main organs’ indexes are shown in Table 2. The spleen index in the CTR group and the IRS-661-treated group was lower than that in the model control group (*p* >0.05).

### 2.3. The Treatment Attenuated ANA Antibodies in the IRS-661-Treated and IRS-869-Nanoflower-Treated Groups and Significantly Reduced dsDNA Antibodies in the IRS-661-Nanoflower-Treated Group

**Figure 2:**
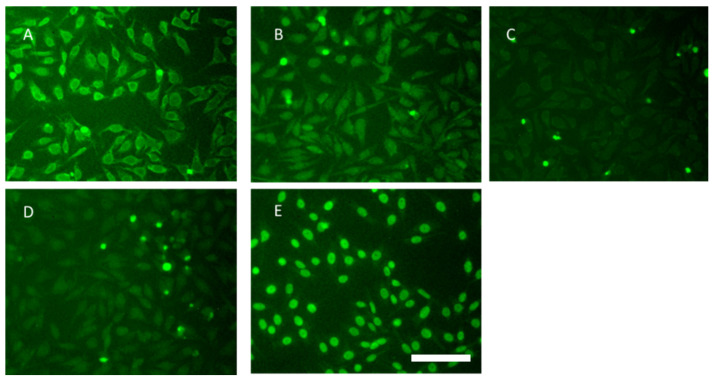
Fluorescence images of ANA tests. (**A**). Model group; (**B**). CTR-nanoflower-treated group; (**C**). IRS-661-nanoflower-treated group; (**D**). IRS-869-nanoflower-treated group; (**E**). IRS-954-nanoflower-treated group. Scale bar: 50 μm.

ANA results are shown in Figure 2 via indirect immunofluorescence ANA detection. The model control group and the IRS-954-nanoflower-treated group appeared to have stronger fluorescence. The model control group was mainly classified as a cytoplasmic, nuclear ANA pattern. The cytoplasmic pattern with dense fluorescence suggested that it was mainly anti-ribosomal antibodies. The IRS-954-nanoflower-treated group showed a mainly homogenous and speckled pattern, suggesting that the antibodies were mainly composed of SLE-characteristic anti-dsDNA antibodies and anti-RNP/Sm antibodies. The fluorescence intensity of nanoflower-treated groups was decreased, except for IRS 954. The CTR-treated group showed lower cytoplasmic staining fluorescence than the model control group. The weak fluorescence intensity of the IRS-661-nanoflower-treated group and the IRS-869-nanoflower-treated group suggested remarkable amelioration effects after treatment. Both were mainly cytoplasmic nuclear ANA patterns with rare fluorescence (as shown in Figure 2).

**Figure 3:**
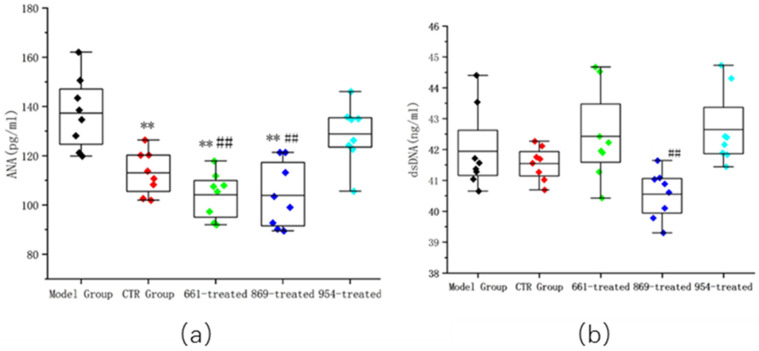
ANA and anti-dsDNA antibody titers. (**a**) Serum levels of ANA in serum; (**b**) serum levels of anti-dsDNA titer in serum. **: Compared with the model group, *p* < 0.01; ##: compared with the 954-treated group, *p* < 0.01.

The serum levels of ANA and anti-dsDNA antibodies were further quantitatively detected (shown in the left panel of Figure 3). The ANA level of the CTR-treated group was 113.01 ± 8.81 pg/mL, and the ANA levels in the IRS-661-nanoflower-treated group and IRS-869-nanoflower-treated group were 104.06 ± 9.25 pg/mL and 103.85 ± 13.30 pg/mL, respectively, which were significantly lower than those in the model group (*p* < 0.01), indicating the alleviation effect of the nanoflower in vivo. However, although the ANA level of the IRS-954-nanoflower-treated group decreased to some extent, there was no significant difference compared with the model group. Even when compared with the IRS-661-nanoflower-treated group and the IRS-869-nanoflower-treated group, the ANA level of the IRS-954-nanoflower-treated group was significantly higher than these two groups (*p* < 0.01).

The results of the anti-dsDNA antibody showed that the level of the IRS-869-nanoflower-treated group was significantly reduced, 40.55 ± 0.77 pg/mL, significantly lower than that in the IRS-954-nanoflower-treated group (*p* < 0.01). It was also lower than that of the model group (*p* > 0.05). These results are shown in the right panel of Figure 3.

### 2.4. Proteinuria Was Down-Regulated in All the Nanoflower-Treated Groups, Especially IRS-661-Treated and IRS-869-Nanoflower-Treated Groups

**Figure 4:**
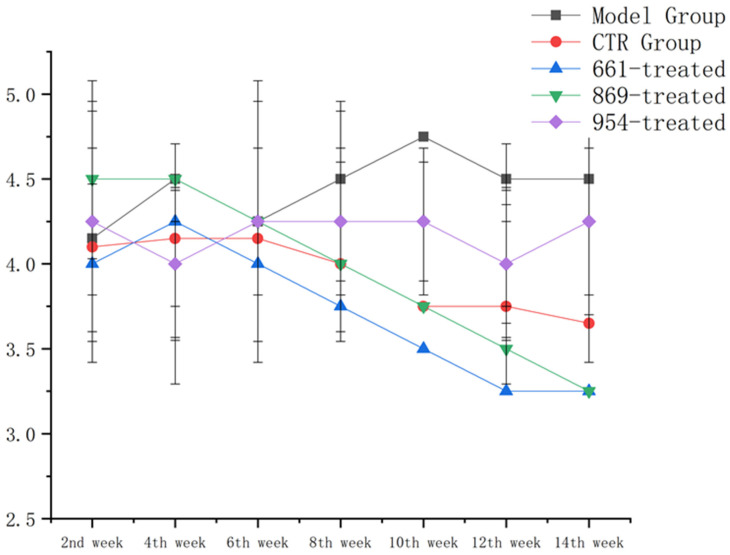
Proteinuria value of each group in different weeks.

The urine protein of nanoflower treatment groups was decreased after treatment, as shown in Figure 4. Conversely, model mice gradually developed exacerbated kidney disease, with oliguria and proteinuria. The urinary protein in the IRS-869-nanoflower-treated group and the IRS-661-nanoflower-treated group were significantly down-regulated, indicating that their renal function was gradually ameliorated.

### 2.5. Lupus Nephritis Was Ameliorated after Nanoflower Treatment but Not in the IRS 954 Group

**Figure 5:**
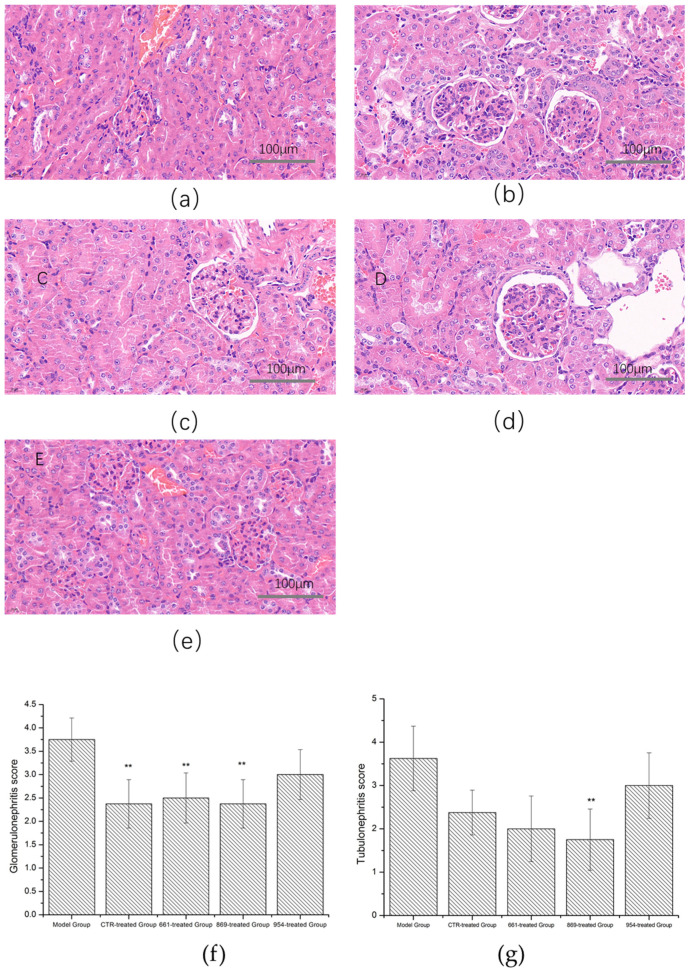
Renal pathological sections examination. (**a**). Model control group; (**b**). CTR-nanoflower-treated group; (**c**). IRS-661-nanoflower-treated group; (**d**). IRS-869-nanoflower-treated group; (**e**). IRS-954-nanoflower-treated group; (**f**). glomerulonephritis score; (**g**). tubular nephritis score (**: compared with the model control group, *p* < 0.01).

The mouse kidneys of nanoflower-treated groups were subjected to H&E staining and pathological examination. Lupus nephritis in all nanoflower-treated groups except for IRS 954 was ameliorated. However, the model group showed diffuse lupus nephritis (as shown in Figure 5).

The images of the kidneys in the model group showed an accumulation of eosinophilic substances with thickening of the basement membrane and increasing mesangial matrix, irregular shape of the glomerulus, and swelling and proliferation of intraglomerular cells and mesangial cells. The kidney images of the IRS-954-nanoflower-treated group were similar to the model group, with a large number of eosinophils compressing capillaries and infiltration of lymphocytes. The glomerulonephritis in the CTR-treated group was ameliorated, and the glomerular tubules were still infiltrated by lymphocytes, but the shape of the glomeruli was relatively regular. Lupus nephritis both in the IRS-661-nanoflower-treated group and the IRS-869-nanoflower-treated group was alleviated significantly. The lupus nephritis inflammation was localized with reduced infiltration, and the morphology of glomeruli and tubules was more regular.

The histologic glomerulonephritis score showed an alleviation effect in the nanoflower-treated groups. The glomerulonephritis scores were significantly reduced in the IRS-661-nanoflower-treated group, IRS-869-nanoflower-treated group, and CTR-treated group compared with the model group, and tubulointerstitial nephritis was also reduced (*p* < 0.01 compared with the model control group, as shown in Figure 5f,g).

### 2.6. Immune Complexes Reduced in the CTR-Treated-, IRS-661-Treated-, and IRS-869-Nanoflower-Treated Groups

**Figure 6:**
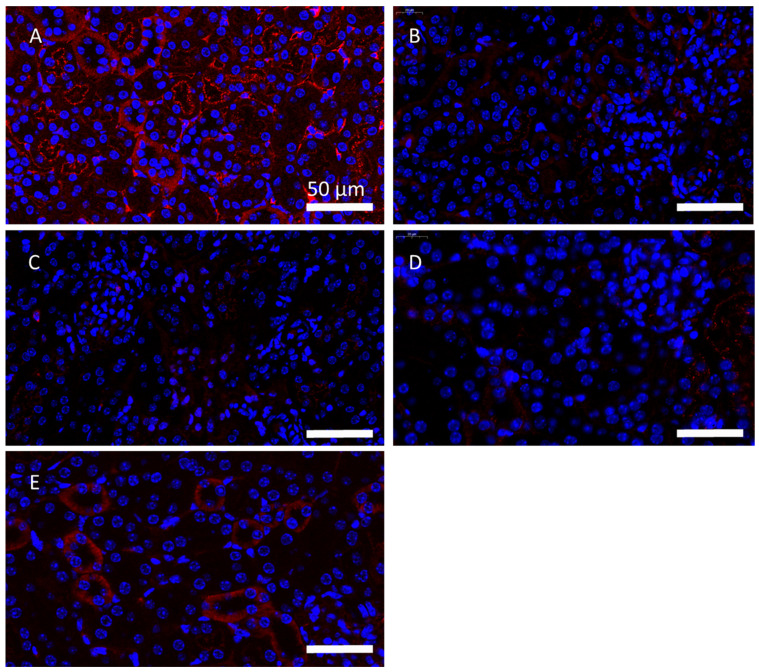
Renal immune complex examination. (**A**). Model group; (**B**). CTR-nanoflower-treated group; (**C**). IRS-661-nanoflower-treated group; (**D**). IRS-869-nanoflower-treated group; (**E**). IRS-954-nanoflower-treated group. Scale bar: 50 μm.

In the model group and the IRS 954 nanoflower group, moderate-to-strong intense immune complexes of fluorescence were observed, with a granular fluorescence distribution, accompanied by the distribution of glomerular capillary loops, generally reflecting the glomerular outline and suggesting the deposition of immune complexes in the glomerulus. The fluorescence of immune complexes in the CTR-nanoflower-treated group, the IRS-661-nanoflower-treated group, and the IRS-869-nanoflower-treated group showed weak to mild fluorescence (shown in Figure 6).

### 2.7. The Serum Level of IL-17 in IRS-661-Treated and IRS-869-Nanoflower-Treated Groups Significantly Reduced; IFNα Levels Decreased Markedly in All Nanoflower-Treated Groups

The serum levels of IL-17 and IFNα in the IRS-661-nanoflower-treated group and IRS-869-nanoflower-treated group were significantly reduced. IL-17 levels were 2.67 ± 0.24 pg/mL and 2.80 ± 0.25 pg/mL, respectively (*p* < 0.05 compared with the model group; *p* < 0.05 compared with the 954-treated group).

**Figure 7:**
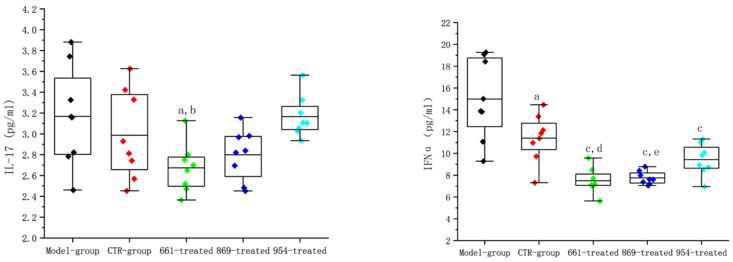
Interferon-α and IL-17 contents in mice serum. a: Compared with the model group, *p* < 0.05; b: compared with the 954-treated group, *p* < 0.05; c: compared with the model group, *p* < 0.01; d: compared with the CTR group, *p* < 0.01; e: compared with the CTR group, *p* < 0.05.

The IFNα levels decreased obviously in all the nanoflower-treated groups (as shown in Figure 7), especially the IRS-661-nanoflower-treated group (compared with the model group, *p* < 0.01; compared with the CTR group, *p* < 0.01). The level of IFNα in the IRS-869-nanoflower-treated group was reduced to 7.75 ± 0.60 pg/mL, which was extremely down-regulated from the model group and the CTR-nanoflower-treated group. The IRS-954-nanoflower-treated group was also significantly lower than the model control group (*p* < 0.05).

## 3. Discussion

Systemic lupus erythematosus is a multifactorial autoimmune disease, and the mechanisms behind it are not yet fully understood [14]. Lupus-susceptible individuals initiate an immune activation, and necrotic cells release self-nucleic acid fragments to activate endosomal nucleic acid sensors. Plasmacytoid dendritic cells (pDCs) activate and secrete interferon α, which in turn stimulates B cells to secrete autoantibodies, and the elevated autoantibodies further accelerate abnormal immune activation in vivo [15]. The immune complex deposits in the skin, kidney, and other tissues, resulting in autoimmune symptoms. Toll-like receptors (TLRs) can recognize specific nucleotide sequences in the CpG oligonucleotides of bacteria and other prokaryotes to activate pDC. Therefore, specific inhibition of TLRs is considered an important strategy for the treatment of SLE.

Inhibitory ODNs showed immunosuppressive effects with modification on the motifs of CpG or backbones. Their inhibitory biological activity was enhanced by the addition of G bases to form G-3-dimensional stacks or by replacing the phosphodiester backbone with the phosphorothioate backbone against nuclease degradation [16]. The repeating core motifs exhibited spatial folding, and the prolonged backbone of the DNA nanoflower was predicted to be beneficial for TLR recognition. Moreover, native phosphodiester (pO) avoided the potential risk of degradation products of phosphorothioate (pS).

Studies have shown that the level of IFN-α in lupus patients is increased and positively correlated with the disease activity of SLE, and the main source of IFN-α is pDCs [17]. pDC recognizes unmethylated CpG and anti-dsDNA antibody immune complexes through Toll-like receptor 9 (TLR9) and activates the production of IFN-α [18]. Furthermore, TLR7 is also believed to bind to RNA–self-antigen complexes, which are involved in the immune response [19]. Thus, the roles and the relationship between TLR7 and TLR9 in lupus immune signaling have gradually received increasing attention and have been investigated.

ODNs containing specific inhibitory oligodeoxyribonucleotide sequences could inhibit DNA-induced immune activation, such as bacterial infection [20]. Moreover, it was found that ODNs alleviated lupus disease in spontaneous NZB × NZW F1 mice and reduced IFN-α levels [13]. The alleviation effect of inhibitory oligonucleotides of TLR9 has been confirmed [21], and TLR7 has also verified that it is one of the target molecules for lupus pathology [22]. These ODN inhibitors were initially shown to inhibit TLR9 signaling and were later found to inhibit immune responses to TLR7 ligands [23,24]. Sean et al. knocked out TLR7 and TLR9 of MRL/lpr, respectively, and found that TLR9^−/−^ mice had aggravated autoimmune disease, more active lymphocytes and plasmacytoid dendritic cells, and increased serum IgG and IFN-α. In contrast, TLR7^−/−^ mice had decreased lymphocyte activation and decreased serum IgG. Although TLR7 and TLR9 co-express on pDCs and B cells and share downstream signaling pathways, whether TLR7 and TLR9 signaling have different roles in immune regulation in lupus’ pathogenic mechanisms is still under investigation. We selected three representative types of INH-ODN sequences in this study—namely, IRS 661, which specifically bound to TLR7; IRS 869, which was an inhibitory sequence of TLR9; and IRS 954, which was considered a dual antagonist to both TLR7/9—to investigate their immunosuppressive effects in lupus-model mice.

Researchers generally believe that TLR7 plays a pathological role in SLE [25]. However, the role of TLR9 seems to be more complicated. In this paper, when TLR7 or TLR9 was blocked alone, the IRS-661-nanoflower-treated group and the IRS-869-nanoflower-treated group showed better amelioration of lupus symptoms. The IRS 661 sequence has five palindromic sequences with evenly distributed “TGC” [13]. Lenert et al. believed that IRS 661 specifically bound to TLR7, relying on its special structure and the backbone replaced phosphodiester with phosphorothioate for anti-nucleic acid enzymatic degradation [10]. The IRS-661-nanoflower-treated group in our study showed a very significant immunosuppressive effect, decreasing autoantibodies and alleviating lupus nephritis in the lupus model. Due to their special structure, nanoflowers can be gradually degraded by nucleases from multiple free ends. Moreover, their palindromic structure helps them form more compactly with the phagocytosis of lysosomes. These are considered crucial factors beneficial for immune regulation. The ODN sequence of IRS 869 is an improved TLR9 inhibitor. It is characterized by a modified backbone to delay degradation by nucleases. In this study, due to the rolling circle amplification, the backbone of nanoflowers was greatly extended. The significant immunosuppressive effect of the IRS 869 nanoflower indicated that the extended skeleton plays a helpful role in the anti-degradation as a TLR9 inhibitor. On the other hand, the immunomodulatory effect of the IRS 869 nanoflower may be related to its spatial folding. Multiple G base repeats or “TTAGGG” motifs formed G4-stacks in space, which was beneficial to binding to TLR9 [16]. This nanoflower increased the combination of G-tetrads into multiple G4 spatial thresholds. Therefore, the recognition and binding ability of the IRS 869 nanoflower to TLR9 was greatly enhanced. However, the mice in the IRS-954-nanoflower-treated group did not show obviously attenuated effects on lupus manifests, although ODN IRS 954 was believed to be a dual antagonist that could bind to both TLR7 and TLR9. The production of autoantibodies relied on TLR9 signals in the spontaneous lupus mouse model [26], but TLR9 deletion aggravated the mortality and renal injury of TLR9^−/−^ in lupus-susceptible mice [11]. It was speculated that the possible reason for this was TLR9^−/−^-enhanced TLR7 signaling, which further aggravated lupus disease [25]. Conversely, TLR7 deletion completely suppressed lupus disease progression in TLR9^−/−^ mice. IRS 954 simultaneously exerting TLR7/9 signaling showed weak down-regulation of dsDNA IgG2a, dsDNA IgG2b, and Smith antigen autoantibodies, but it was not helpful in lupus nephritis. Thus, the alleviating effect of IRS 954 was far lower than IRS 661 [27]. TLR9 antagonism provided by IRS 954 “neutralized” the inhibitory effect of IRS 661 on TLR7-mediated production of anti-dsDNA IgG2a, IgG2b, and anti-Sm IgG. It appeared that TLR7 signaling played a more dominant role in immunoregulation.

We found that the nuclear ANA pattern of the IRS-954-treated group differed from those in other groups. The IRS-954-treated group showed homogeneous and speckled nuclear ANA patterns, while the other groups mainly exhibited cytoplasmic nuclear ANA patterns. This indicated that there were mainly anti-dsDNA antibodies and anti-RNP/Sm autoantibodies in the IRS-954-treated group, which was consistent with previous reports. These results further suggested that the concomitant blockade of TLR9 with the IRS 954 nanoflower neutralized the effect of TLR7 blockade but had neither additive nor opposing effects on autoimmune kidney injury [27]. Hence, we supposed that the effect of the deficient TLR9 gene might be due to different mechanisms instead of the targeted blockade of the TLR9 signal. In the TLR9-knockout model, TLR7 may be overstimulated due to its ligand surplus [28]. In contrast, there were both anti-dsDNA antibodies and anti-RNP/Sm autoantibodies in the IRS-954-nanoflower-treated group, revealing the complexity of the interaction between TLR9 and TLR7 signaling in IRS-954-treated mice. This provided us with a new perspective on the possible switching of the autoantibody repertoire when IRS 954 was co-acting with TLR7/9 signaling in vivo.

It had been clinically found that there were high levels of IFN-α in the plasma of SLE patients. The pDCs were the main source of IFN-α in vivo [29]. Type I interferon rapidly elevated after viral infection, which involved the recognition and combination of nucleic acids via intracellular nucleic acid receptors or Toll-like receptors [30]. Type I interferon, mainly IFN-α, was involved in the pathogenesis of SLE. An intimate correlation between an elevated serum IFN-α in patients with active lupus and microbial infection TLR7/9, pDCs, and IFN-α were believed to interact with each other. Studies found a positive correlation between an increase in secretion in IFN-γ and IFN-α in SLE patients. Further, it confirmed that IFN types I and II partially share signal pathways and target genes. Moreover, IFN-γ is indispensable for the TLR7-promoted development of autoreactive B cells and systemic autoimmunity [31]. Due to microbial stimulation, TLR7/9 signal activation promoted pDC to secrete IFN-α, promoting B-cell differentiation and up-regulating the expression of TLR7 and MyD88. TLR7 synergistically promotes the differentiation of ABC/DN2 B-cell subsets with the help of cytokines such as IFN-γ and IL-21. Type Ⅰ IFN and TLR7 enhance autoantibody production from transitional B cells [32]. These IFN interactions help coordinate the specific functions of SLE immunopathogenesis [33] and self-reactivity with DNA or RNA immune complexes was enhanced [29]. Our study showed that IFN-α decreased after nanoflower treatment, indicating that nanoflowers could specifically inhibit the secretion of IFN-α and have an immunosuppressive effect on TLR7/9 signaling in vivo, which helped alleviate lupus disease.

Particularly, the alleviation of lupus disease in the CTR-nanoflower-treated group revealed that the special sequence might not be the only factor influencing the immune-regulation effect. CTR, as a nonspecific meaning sequence of nanoflower, showed a significant decrease in IFN-α after treatment (compared with the model group, *p* < 0.05). This indicated that the inhibitory effect depended not only on a special motif but a sufficient sequence or backbone also exerted immunosuppressive effects [34].

Th17 cells induced neutrophil-mediated glomerulonephritis, and both Th1 and Th17 effector cells were involved in glomerulonephritis and exacerbated the severity [25]. Th17 cells secreted IL-17 by stimulating endogenous renal cells and immune cells to synthesize inflammatory cytokines. It finally induced renal inflammation and fibrosis. Serum levels of IL-17 in lupus patients were significantly correlated with lupus nephritis biopsy grades. Meanwhile, IL-17 levels in vivo were closely associated with autologous antibody titers [35]. This study found that the level of IL-17 was down-regulated after DNA nanoflower treatment, especially in the IRS-661-nanoflower-treated group. The serum level of ANA was also significantly reduced, as well as lupus nephritis amelioration. Moreover, the serum level of type I interferon reduced as IL-17 decreased, which is consistent with the current research on synergy between IFN-α and IL-17. The glomerular deposition of IgG and immune complexes was reduced, and decreased expression of Th1-related pro-inflammatory factors in the kidney may be the reason [36]. Thus, IFN-α and Th17 are suggested as mechanisms co-regulating the pathogenesis of lupus disease [37].

## 4. Materials and Methods

### 4.1. Preparation and Characterization of the DNA Nanoflower

The core sequences of INH-ODNs with a confirmed immunosuppressive effect on TLR7 or TLR9 were selected. The sequences are shown in Table 3. IRS 661 (5′-TGCTTGCAAGCTTGCAAGCA-3′), IRS 869 (5′-TCCTGGAGGGGGTTGT-3′), and IRS 954 (5′-TGCTCCTGGAGGGGTTGT-3′) were added to T4 ligase 5′-TCACCATAGTCCTTGTCATCAC-3′ as the connecting sequence for circular amplification, respectively. The nanoflower of random sequence, named CTR, was prepared as control group. For CTR nanoflowers, a randomly generated DNA sequence (5′-AGGTTGACTTCCACTCTACTCAG-3′) was adopted from the online random DNA generation tool (https://www.genscript.com/sms2/random_dna.html (accessed on 5 January 2020)). The preparation steps of CTR nanoflower were the same as those in INH-ODNs nanoflowers.

**Figure 8:**
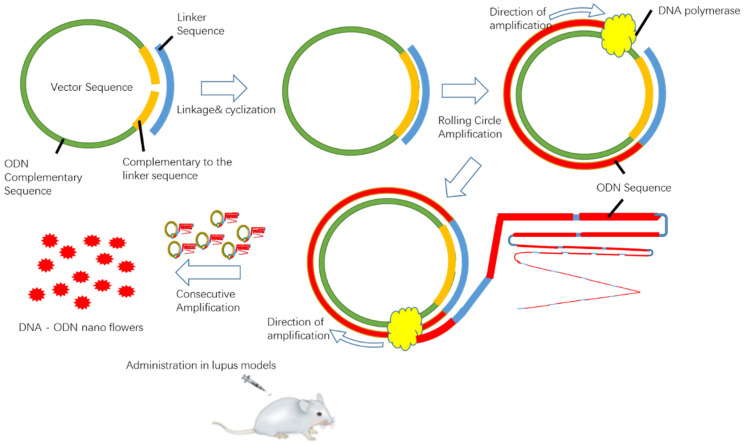
Schematic diagram of the preparation process of DNA nanoflowers.

A total of 50 μL of the cyclization reaction product was added to a 400 μL rolling circle amplification reaction system (the system was 1× reaction buffer, 1 mM dithiothreitol, 10 U phi29 DNA polymerase, 10 mM dNTP) for 16 h at 37 °C. The product was fully dispersed at 65 °C to inactivate DNA polymerase for 10 min. It was washed with sterile water, centrifuged at 20,000× *g* to separate DNA nanoflowers, and stored at 4 °C for later use (the preparation process is shown in Figure 8).

The ultrastructure of the prepared DNA nanopolymer was observed by scanning electron microscope (S-4800, Hitachi, Ibaraki, Japan), and the particle diameter was counted. Electron microscope resolution: 2.0 nm, accelerating voltage 1 kV, WD = 1.5 mm.

### 4.2. Animal Grouping and DNA Nanoflower Administration

Female MRL/lpr mice were purchased from Slack Laboratory Animal Co., Ltd. (Shanghai, China) and were raised in the SPF facility of the Experimental Animal Center of Soochow University. The animal protocol was approved by the animal ethical committee at Soochow University in China. MRL/lpr mice were administrated when they reached 24 weeks old. The animals were divided into 5 groups (8 animals in each group): model control group (model group), CTR nanoflower control group (CTR-treated group), IRS-661-nanoflower-treated group (IRS-661-treated group), IRS-869-nanoflower-treated group (IRS-869-treated group), and IRS-954-nanoflower-treated group (IRS-954-treated group). All the groups were subcutaneously injected with either saline or prepared nanoflower twice a week (10 μg/250 μL/mice/per time) for a total of 14 consecutive weeks of treatment.

At the end of the experiment, the body weight of the animal was weighed, and the main organs of the animal, including heart, liver, spleen, lung, and kidney, were collected and weighed immediately after euthanasia. To calculate the organ index, the formula used was organ index = (organ weight (g)/body weight (g)) × 100%.

### 4.3. ANA Analysis

Serum was collected before injection and once a month after injection and stored at −80 °C for later use. Mouse serum ANA was detected by indirect immunofluorescence method according to the instructions of the ANA detection kit (Suzhou Haooubo Biomedical Co., Ltd. Suzhou, China). Serum was diluted at 1:1000 and then incubated with Hep-2 cell slide reaction area for 30 min at room temperature and observed under a fluorescence microscope. The results were diagnosed as follows: nonvisible fluorescent karyotype at serum dilution 1:1000 was negative; visible fluorescent karyotype at serum dilution of 1:1000 was positive.

### 4.4. Quantification of ANA and Anti-dsDNA Antibodies 

The animal serum was diluted at 1:100 with ANA detection kit diluent (Wuhan Huamei Bioengineering Co., Ltd. Wuhan, China). The standard substance and the tested samples were added to the microplate plate, and the absorbance value was measured at a wavelength of 450 nm. According to the standard curve formula, the animal serum ANA level was calculated.

The same method was used as described above to quantify the anti-dsDNA antibody level at a 1:60 dilution of serum.

### 4.5. Detection of Proteinuria

Mice urine protein was measured by Albustix urine protein test. The urine from mice was measured biweekly after the administration. The urine protein concentration marked (−), (±), (+), (++), (+++), (++++) according to the illustration of color development in the reaction zone. (−) was recorded as 0 points, and (±), (+), (++), (+++), and (++++) were marked as 1, 2, 3, 4, and 5 points, respectively.

### 4.6. Histological Evaluation

The euthanized mice kidneys were immediately fixed with 10% formaldehyde, dehydrated, embedded, and stained with H&E. Then, the pathological characteristics of slice were diagnosed under light microscope. The pathological changes, such as lymphocyte infiltration and fibrous tissue hyperplasia in the interstitium, were observed.

Renal pathological damage and hyperplasia were scored by semi-quantitative scoring: 0 points for normal, 1 point for 35–40 cells per glomerular cross-section (c/gcs), and 2 points rated for mild cell hyperplasia (41–50 c/gcs). Moderate hyperplasia (51–60 c/gcs) or some proliferative pathological changes or vacuolar degeneration was rated as 3 points. Severe hyperplasia (>60 c/gcs) with severe cell necrosis and crescent formation was scored as 4 points. The renal tubular scoring standard was based on the degree of cortical tubular enlargement, atrophy, or necrosis. It was divided into 1 point, =<10%; 2 points, 10–25%; 3 points, 26–75%; 4 points, =>75%.

### 4.7. Immune Complex (IC) Detection

The kidneys of the animals were collected and fixed with 10% formaldehyde and dehydrated in ethanol. They were embedded in paraffin, sectioned, and then stained with peroxidase-labeled rabbit anti-mouse IgG polyclonal antibody (Jackson ImmunoResearch Inc., West Grove, PA, USA). After incubation at 37 °C for 30 min in the dark, unbound antibodies were washed away. Nuclei were counterstained with DAPI (4′,6-diamidino-2-phenylindole). The coverslips were observed under a fluorescence microscope (ultraviolet excitation wavelength 330–380 nm; emission wavelength 420 nm).

### 4.8. Quantitative Detection of the Level of Cytokines IFNα and IL-17

The final serum at euthanasia was diluted to 1:10. The optical density of each well was measured at a wavelength of 450 nm according to the mouse IFNα ELISA enzyme-linked immunosorbent assay kit (Wuhan Huamei Biological Engineering Co., Ltd. Wuhan, China). The level of mouse IFNα of each group was calculated based on the standard curve formula.

Similarly, the optical density was measured according to the mouse IL-17 ELISA enzyme-linked detection kit (Wuhan Huamei Biological Engineering Co., Ltd.) at 450 nm, and the serum IL-17 level of each group was assessed.

### 4.9. Statistical Processing

Origin 8.5 software (OriginLab Corporation, Northampton, MA, USA) was used for statistical analysis of data; experimental data are expressed as mean ± standard deviation (SD). The difference between two values was regarded as statistically significant (*p* < 0.05) when the confidence intervals did not overlap.

## 5. Conclusions

In summary, a novel DNA assembly method was used to form highly compressed nanoflowers. In this study, its immunosuppressive effect as a TLR7 or TLR9 antagonist was investigated in a lupus mouse model. The results showed that both of the nanoflowers, as specific antagonists for TLR7 and TLR9, could decrease the level of autoantibodies, reduce cytokine secretion, and alleviate lupus nephritis in mice. It was hypothesized that the specific motifs and the extended backbone were beneficial for the blockade of TLR7 or TLR9 signaling. However, the IRS 954 nanoflower, the TLR7 and TLR9 dual antagonist, did not have additive or opposing effects on lupus nephritis but only showed a decrease in serum IFNα, suggesting that TLR7 and TLR9 antagonists may have a competition mechanism or signal-dependent switching relationship under simultaneous action. The combined blockade of TLR7 and TLR9 had fewer additive effects. These data support the concept that endogenous ligands of TLR7 and TLR9 contribute to the pathogenesis of autoantibody production and autoimmune tissue injury in SLE and provide a new strategy for exploring lupus therapeutic agents.

## Figures and Tables

**Table 1:** Weight of body and main organs in each group.

Groups	Body Weight	Heart	Liver	Spleen	Lung	Kidney
Model Group	35.64 ± 5.30	0.18 ± 0.03	1.93 ± 0.42	0.64 ± 0.17	0.26 ± 0.03	0.57 ± 0.13
CTR-treated	36.49 ± 1.01	0.19 ± 0.03	2.31 ± 0.60	0.60 ± 0.08	0.31 ± 0.07	0.54 ± 0.13
IRS-661-treated	36.39 ± 1.58	0.18 ± 0.03	2.11 ± 0.19	0.61 ± 0.10	0.24 ± 0.03	0.55 ± 0.05
IRS-869-treated	37.33 ± 4.26	0.19 ± 0.03	2.18 ± 0.25	0.71 ± 0.19	0.28 ± 0.04	0.58 ± 0.07
IRS-954-treated	34.97 ± 1.99	0.16 ± 0.02	2.06 ± 0.32	0.62 ± 0.20	0.24 ± 0.03	0.49 ± 0.07

**Table 2:** Weight ratio of each group.

Groups	Heart Index	Liver Index	Spleen Index	Lung Index	Kidney Index
Model Group	0.52 ± 0.09%	5.36 ± 0.48%	1.76 ± 0.29%	0.76 ± 0.18%	1.58 ± 0.19%
CTR-treated	0.51 ± 0.07%	6.29 ± 0.47%	1.64 ± 0.18%	0.84 ± 0.17%	1.47 ± 0.32%
IRS-661-treated	0.49 ± 0.08%	5.81 ± 0.64%	1.66 ± 0.24%	0.65 ± 0.10%	1.52 ± 0.17%
IRS-869-treated	0.51 ± 0.11%	5.86 ± 0.59%	1.88 ± 0.31%	0.75 ± 0.19%	1.59 ± 0.33%
IRS-954-treated	0.46 ± 0.06%	5.88 ± 0.74%	1.77 ± 0.56%	0.68 ± 0.06%	1.39 ± 0.15%

**Table 3:** Study target sequence.

ODN	Deoxynucleotide Sequence
IRS 661	5′- GACTATGGTGACTCAA**TGCTTGCAAGCTTGCAAGCA**GACTGTGATGACAAG-3′
IRS 869	5′-GACTATGGTGACTC**TCCTGGAGGGGTTGT**GCAACTGTGATGACAAG-3′
IRS 954	5′-GACTATGGTGACTC**TGCTCCTGGAGGGGTTGT**GCAACTGTGATGACAAG3′
CTR	5′-GACTATGGTGACTCAGGTTGACTTCCACTCTACTCAGACTGTGATGACAAG-3′

The bold part is the core target sequence; meanwhile, the underlined part is the circularization link sequence.

## Data Availability

The data of current study are available from the corresponding author on reasonable request.
